# Otosclerosis

**Published:** 1909-06-12

**Authors:** 


### Otology.

### OTOSCLEROSIS.

There is a very important, because very common, form of deafness, the real nature of which has only recently been understood, and which appears to be not yet generally recognised by the profession at large. This affection is known as "otosclerosis," and, although not a correct description of its pathology, the title has been generally adopted and will doubtless be retained. This disease is marked by definite characteristics which render its recognition in uncomplicated cases a comparatively easy matter. The principal features are increased bone-conduction and a negative Rinne's test, signs found in cases of middle-ear catarrh, combined with a practically normal drum-membrane and a patent Eustachian tube, inflation through which causes no improvement.

### Pathology.

The Eustachian tube, the middle ear, and the membrana tympani are normal; the pathological changes which are the cause of the affection occur in the neighbourhood of the stapes and fenestra ovalis. The fundamental change is the absorption of the normal tissues and their replacement by a new growth of spongy bone. The stapes becomes fixed in the fossa ovalis by extension of bone along the foot-plate, or by bony bridges from the wall of the labyrinth to its crura. In advanced cases the entire fossa ovalis may become filled with spongy bone, and sometimes bony bosses protrude into the interior of the labyrinthine capsule. As time goes on the newly formed spongy bone becomes gradually denser and less distinguishable from the normal bone adjacent to it.

### Ætiology.

It may be said at once that this is not a true inflammatory process, and that there is a complete absence of signs of inflammation or any history of previous inflammatory disease in these cases. But we are a long way from certainty as to the underlying cause of the process. It may be, as has been suggested by Siebenmann, that it is the final stage of the developmental process, which is normal in all bones except the petrous; numerous remnants of the primary cartilage are to be found in the neighbourhood of the fenestra ovalis, and possibly the conversion of these into bone, by a kind of over-completion of development, may be at the root of the process; for the normal development of the petrous bone is arrested at birth, as it has by then attained its full size. Gray has propounded the theory that the original lesion is a necrosis of the parts followed by aseptic absorption and reposition, this necrosis being due to malnutrition; certain clinical facts seem to favour this view, for anæmia, rheumatism, and pregnancy predispose to the affection, which is nearly always bilateral and far commoner in women.

Otosclerosis is a disease of early adult life and is most often first noticed between the ages of twenty and thirty, and, as just mentioned above, is much more often found in women than in men; the proportion is variously stated, but the average may be fixed at about two to one. Heredity has a very marked influence, and a history of inheritance can be obtained in a large proportion of cases. Of exciting causes, anæmia, pregnancy, and severe exposure to cold appear to be definitely proved to conduce to the affection; and patients suffering from otosclerosis tend to become worse with each successive pregnancy. The disease has also been ascribed to gout, rheumatism, and syphilis, but, perhaps, without sufficient proof. As regards the latter, there is no association whatever between otosclerosis and the ordinary signs of congenital syphilis; acquired syphilis is common in men, whereas otosclerosis is much more often found in women, and antisyphilitic treatment is absolutely without influence on the latter disease.

### Symptoms.

The chief symptom is very gradually increasing deafness. In the final stage it is always bilateral, but at first, and sometimes for a long time, the trouble may be limited to one ear. The progress may be continuous or may be stationary for long periods, alternating with times of more rapid deterioration. Unless the internal ear is also affected the deafness is never absolute, and loud speech can still be heard. Tinnitus is very frequently present and may be extremely severe; in some cases it is constant, in others intermittent. Vertigo is comparatively rare and seldom severe. The phenomenon of hearing better in a noise, known as "paracusis Willisii," is nearly always present; it is an early symptom in otosclerosis and a late one in catarrhal deafness, an observation which supports the long-held opinion of the unfavourable significance of this symptom.

### Physical Signs.

The drum membrane is normal, unless the case is complicated by the presence of catarrhal otitis. Sometimes there can be seen a red reflex shining through the drum and due to hyperæmia of the promontory; this is very characteristic, though by no means always to be seen. The Eustachian tubes are fully patent and inflation generally produces no improvement whatever. Three important signs can be elicited, namely, increase of bone-conduction, a negative Rinne's test, and elevation of the lower tone-limit. Increase of bone-conduction is always present unless the labyrinth has become invaded, which often occurs in the latest stage of the affection, and then the bone-conduction becomes diminished. So also a negative Rinne's test, the tuning-fork heard longer on the mastoid than at the meatus, may become modified by affection of the labyrinth, but by this time the deafness has become extreme. Loss of perception of the lower tones of the scale is a very important sign of fixation of the stapes, and therefore occurs constantly in otosclerosis, though it may also occur late in cases of catarrhal and post-suppurative deafness. Gelle's test for fixation of the stapes may also be used; a tuning-fork is placed on the mastoid while the air in the meatus is compressed with Siegle's speculum, the sound is lessened on compression if the stapes is movable but is unchanged if it is fixed.

### Treatment.

No method of treatment as yet discovered can claim any decided success. Potassium iodide and thyroid extract have their advocates. Phosphorus in doses of 10 minims, gradually increased to 30 or 40, of a $\frac{1}{10}$ per cent. solution in almond oil, prescribed in capsules, has been recommended. Of local measures, the nose and naso-pharynx are usually normal, and treatment of these regions has no effect; inflation is useless in cases uncomplicated by catarrh. In a certain number of cases pneumo-massage, applied by means of an electrically driven air-pump inserted in the meatus, has produced distinctly good results. In most cases it does no good, but it is the only form of treatment which holds out any promise, and is therefore worth a trial if the patient wishes to attempt it after being informed of the problematical nature of the result. It would probably be more often successful if carried out at an earlier stage.

